# Characterisation of MRI Appearance of the Spinal Cord Syrinx in the Whole Spectrum of Chiari Malformation

**DOI:** 10.7759/cureus.100225

**Published:** 2025-12-27

**Authors:** Suman Hela, Utpalendu Das, Sumit Chakraborty, Samir Rana

**Affiliations:** 1 Radiodiagnosis, College of Medicine & Sagore Dutta Hospital, Kolkata, IND; 2 Radiodiagnosis, Institute of Post-Graduate Medical Education and Research and Seth Sukhlal Karnani Memorial Hospital, Kolkata, IND; 3 Radiodiagnosis, Dr. B C Roy Multi Specialty Medical Research Centre, Kharagpur, IND

**Keywords:** cervico-dorsal spinal cord, chiari malformation, lower motor neuron (lmn), syrinx, upper motor neuron (umn)

## Abstract

Chiari malformations constitute a spectrum of hindbrain anomalies characterized by downward herniation of posterior fossa structures through the foramen magnum and are frequently associated with spinal cord syrinx, a fluid-filled intramedullary cavity that can result in progressive neurological deficits. With the advent of MRI, accurate, non-invasive characterization of Chiari malformations and associated syrinx has become possible, particularly in pediatric populations where early detection is crucial. This hospital-based cross-sectional study was conducted between February 2020 and July 2021 at the Institute of Post-Graduate Medical Education and Research and Seth Sukhlal Karnani Memorial Hospital, Kolkata, and included 31 (100%) pediatric patients (<12 years) with suspected or confirmed Chiari malformations. MRI of the brain and spine was performed using a 3.0 Tesla system to evaluate syrinx presence, level, length, morphology, and continuity, while clinical features of upper motor neuron (UMN) and lower motor neuron (LMN) involvement were documented. Results revealed that most patients were female, 65% (N=21), with earlier presentation in Chiari Type II and III cases (<18 months) compared to Type I and 0 (>24 months). Syrinx was present in 43% (N=13) of cases, but its occurrence showed no statistically significant independent association with specific Chiari subtypes (p = 0.4395). The syrinx most frequently involved the cervico-dorsal region (31.4% (N=9)), followed by the cervical (30.4% (N=8)) and dorsolumbar (21.7% (N=7)) segments, and extended beyond 3 cm in length in 55.6% (N=17) of cases. All syrinxes were centrally located and continuous, with no eccentric or skip-pattern cavities observed. Clinically, LMN features were more common (55%, N=17) than UMN signs (45%, N=14). The study concludes that MRI is indispensable for the detailed characterization of Chiari malformations and syrinx, with the cervico-dorsal segment being most commonly affected, syrinx morphology typically central and continuous, and its association with Chiari malformations appearing random rather than subtype-specific. These findings underscore the importance of MRI in early diagnosis, clinical correlation, and surgical planning while also highlighting the need for larger, multicentric studies to clarify pathogenetic relationships and optimize patient management strategies.

## Introduction

Chiari malformations are structural abnormalities of the hindbrain, characterized by herniation of the cerebellar tonsils or other posterior fossa contents through the foramen magnum into the cervical spinal canal. First described by Hans Chiari in 1891, these malformations have since been classified into distinct subtypes (I-V) and the debated Type 0, with varying anatomical and clinical presentations [1]. Chiari Type I, the most common, involves herniation of the cerebellar tonsils, whereas Type II presents with herniation of the brainstem and vermis, typically associated with myelomeningocele and hydrocephalus. Type III involves encephalocele, while Type IV is characterized by cerebellar hypoplasia. In India, Chiari malformations are not uncommon, with an estimated burden exceeding one million cases annually [2].

A frequent complication of Chiari malformations is syringomyelia, or spinal cord syrinx, which is defined as a fluid-filled cavity within the spinal cord parenchyma. 'Syrinx' is a collective term that includes hydromyelia, syringomyelia, syringohydromyelia, and syringobulbia [3]. Syringomyelia is clinically significant because it can lead to progressive neurological deterioration, with manifestations ranging from weakness, spasticity, and hyperreflexia in upper motor neuron (UMN) involvement to flaccid paralysis and muscle wasting in lower motor neuron (LMN) involvement [4, 5]. Sensory impairment and scoliosis are also frequent, particularly in pediatric cases. The prevalence of syrinx in Chiari I malformations has been reported in up to 70% (N=22) of cases, although rates vary depending on diagnostic methods and study populations [6].

The exact pathogenesis of syrinx formation remains debated. Gardner proposed the “water-hammer” theory, wherein cerebrospinal fluid (CSF) pulsations are transmitted into the central canal due to obstruction of the fourth ventricular outlets, resulting in syrinx expansion. Williams advanced the concept of craniospinal pressure dissociation, whereby increased intracranial pressure forces CSF into the central canal due to a ball-valve effect at the foramen magnum [7]. Aboulker and Aubin suggested that obstruction at the foramen magnum impedes CSF absorption, increasing spinal CSF pressure and predisposing to syrinx formation [8]. More recent evidence highlights abnormal CSF flow dynamics at the craniocervical junction as a central mechanism [9].

MRI has revolutionized the assessment of Chiari malformations and syringomyelia. MRI provides high-resolution visualization of the posterior fossa and spinal cord, allowing noninvasive classification, surgical planning, and postoperative evaluation. The diagnostic threshold for Chiari I malformation is typically defined as cerebellar tonsillar descent of ≥5 mm below the foramen magnum on sagittal T2-weighted imaging [10]. However, Mikulis et al. demonstrated that tonsillar position varies with age and proposed age-adjusted criteria: 6 mm in the first decade, 5 mm in the second to third decades, 4 mm in midlife, and 3 mm in the elderly [11]. MRI also enables detailed characterization of syrinx morphology, including level, length, central versus eccentric location, and continuous versus skip pattern. Additionally, cine-phase contrast MRI facilitates dynamic assessment of CSF flow, furthering understanding of syrinx pathophysiology.

Despite advances, the relationship between syrinx morphology and Chiari subtype remains incompletely understood. While syrinx is common in Chiari I malformations, its presence in Types II and III may be confounded by associated anomalies such as myelomeningocele and hydrocephalus [12]. Moreover, whether syrinx characteristics correlate with clinical features or prognosis is still debated, creating a gap in current knowledge.

The present study aims to characterize the MRI appearance of spinal cord syrinx across the spectrum of Chiari malformations in pediatric patients. By analyzing syrinx prevalence, anatomical distribution, length, morphology, and continuity, and correlating these with UMN and LMN clinical features, this study seeks to offer novel perspectives on whether syrinx occurrence is random or subtype-specific. Such work is crucial in India, where the disease burden is high and early diagnosis and management have significant implications for long-term neurological outcomes.

## Materials and methods

Study and design

This was a hospital-based cross-sectional observational study conducted in the Department of Radio Diagnosis at the Institute of Post-Graduate Medical Education and Research and Seth Sukhlal Karnani Memorial Hospital, Kolkata, India. The study was carried out over an 18-month period, from February 2020 to August 2021, after obtaining ethical clearance from the Institutional Ethics Committee (IPGME&R/IEC/2020/379). Written informed consent was obtained from the parents or guardians of all participating children.

Study population

The study population comprised 31 (100%) pediatric patients (<12 years of age) who presented with clinical suspicion or radiological evidence of Chiari malformation. Patients were referred from the Departments of Neurosurgery and Pediatrics for detailed neuroimaging evaluation. Both symptomatic and incidentally detected cases were included. Table 1 shows the inclusion and exclusion criteria for the patients in this study.

**Table 1:** Inclusion and exclusion criteria for the study population. The table summarizes the framework used to define the study cohort, including children under 12 years with MRI-confirmed Chiari malformation (Types 0–III) and those with spinal cord abnormalities requiring syrinx evaluation, provided complete clinical and imaging data were available. Exclusions applied to children older than 12 years, cases with post-traumatic or tumor-related syringomyelia, and scans compromised by motion artefacts, ensuring a uniform and reliable study population.

Inclusion Criteria	Exclusion Criteria
Children aged less than 12 years with MRI features suggestive of Chiari malformation (Types 0–III).	Children older than 12 years.
Patients with associated spinal cord abnormalities requiring syrinx evaluation.	Patients with post-traumatic or tumor-related syringomyelia.
Availability of complete clinical and imaging data.	Children with poor imaging quality due to motion artefacts.

Clinical evaluation

All patients underwent a detailed neurological examination performed by experienced clinicians. Clinical features were categorized into UMN and LMN involvement. Signs included spasticity, exaggerated reflexes, and hypertonia, while LMN signs included hypotonia, muscle wasting, areflexia, and weakness. Associated symptoms such as scoliosis, bulbar features, developmental delays, and chronic headaches were also documented.

Imaging protocol

MRI of the brain and spine was performed using a Signa HDxT 3TMR MRI scanner (GE Healthcare, Chicago, USA). Standard pediatric neuroimaging protocols were applied.

Sequences included sagittal and axial T1-weighted spin echo (SE), sagittal and axial T2-weighted turbo spin echo (TSE), fluid-attenuated inversion recovery (FLAIR), short tau inversion recovery (STIR) for spine screening, gradient echo (GRE) sequences in selected cases, and cine phase-contrast MRI for CSF flow dynamics where indicated.

**Figure 1:**
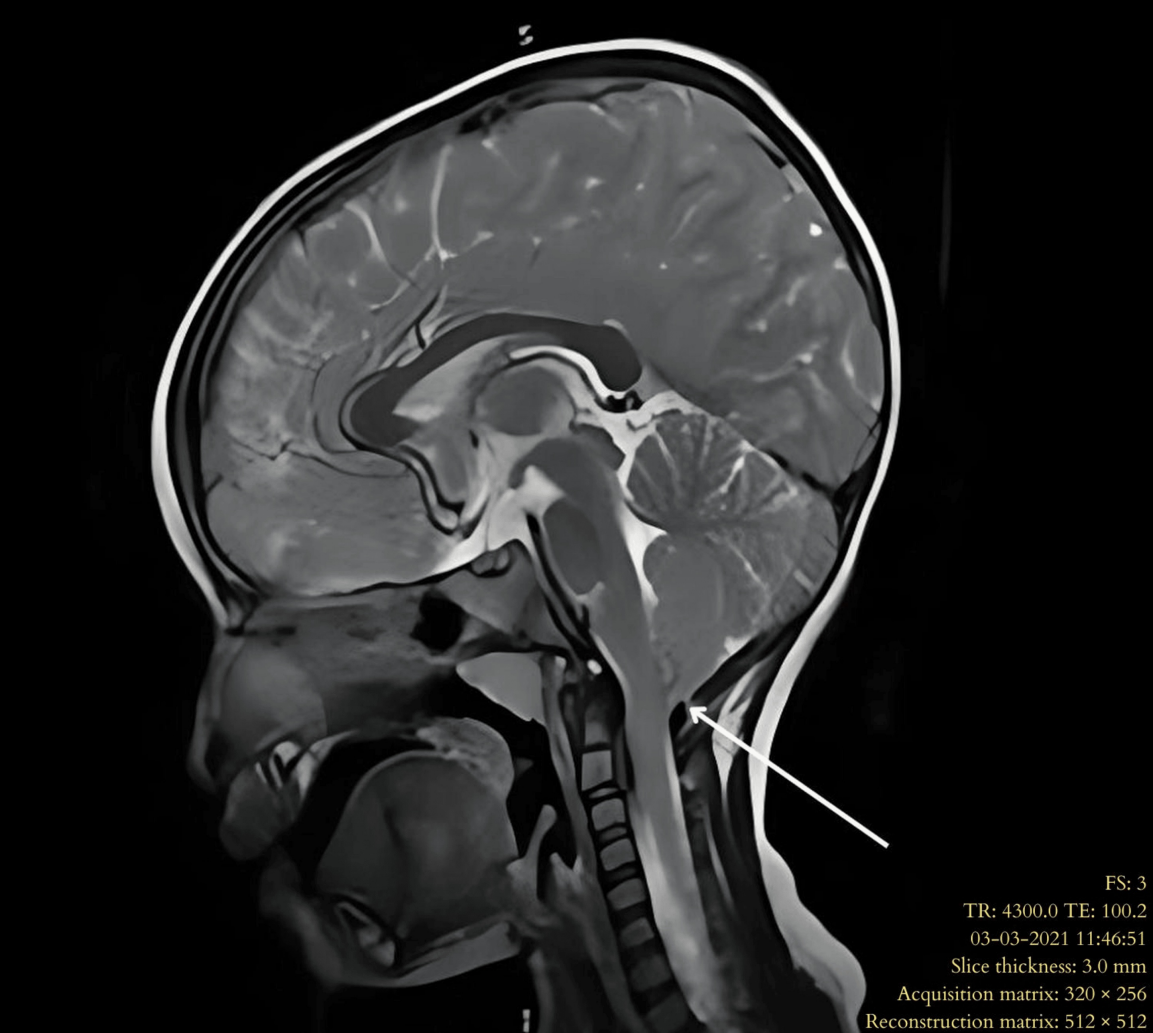
MRI sagittal T2-weighted sequence of the brain in Type 1 Chiari malformation showing a peg-shaped cerebellar tonsil (white arrow) with a crowded foramen magnum.

**Figure 2:**
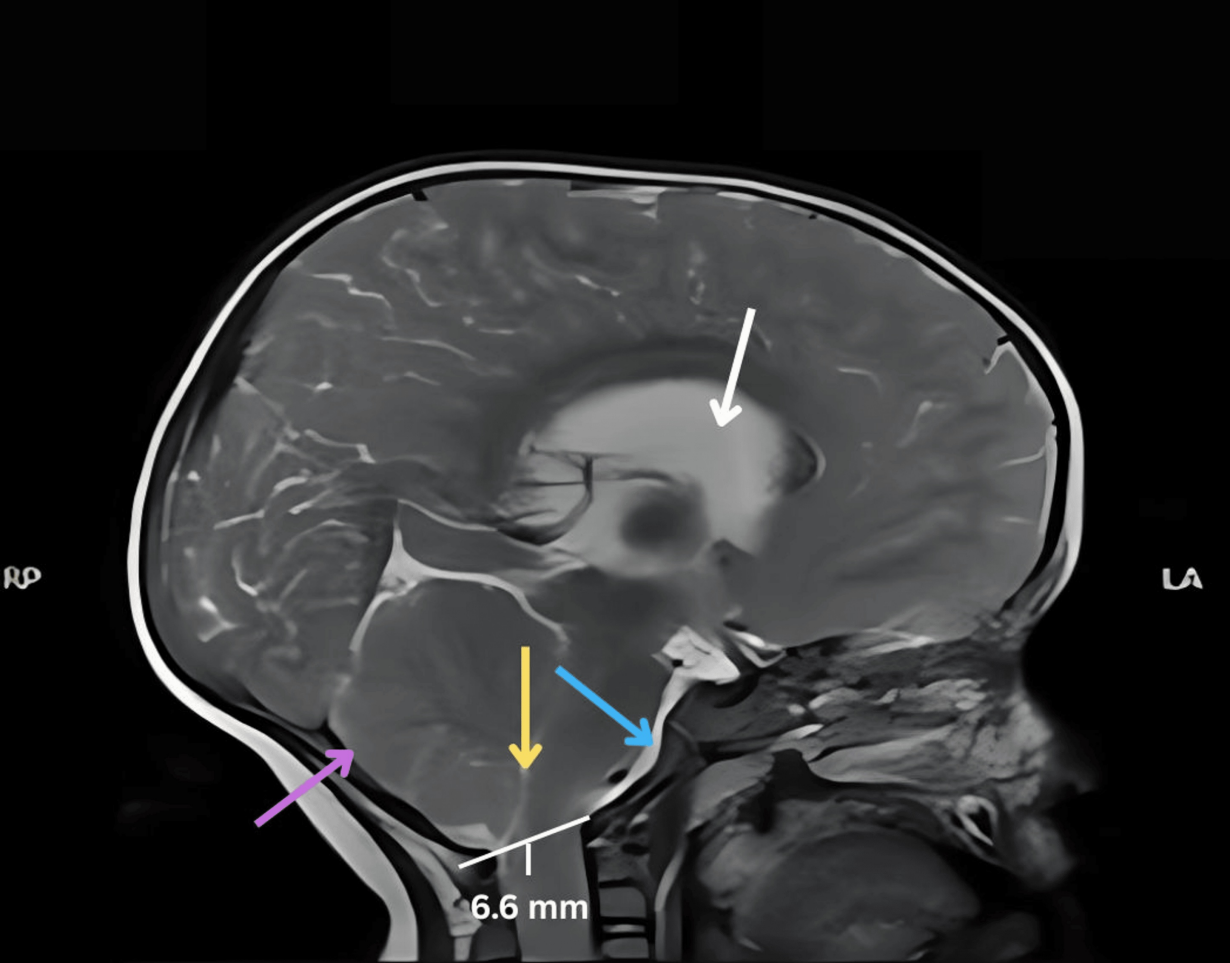
MRI sagittal T2-weighted sequence of the brain in Type 2 Chiari malformation showing supratentorial ventriculomegaly (white arrow). The bony posterior fossa appears small and shallow with a dysplastic concave clivus (blue arrow). The posterior fossa is crowded with effacement of premedullary and retrocerebellar cerebrospinal fluid spaces (purple arrow). The fourth ventricle is slit-like and obliterated (yellow arrow). Herniation of the cerebellar tonsil is seen beyond the opisthion–basion line, measuring a distance of 6.6 mm.

**Figure 3:**
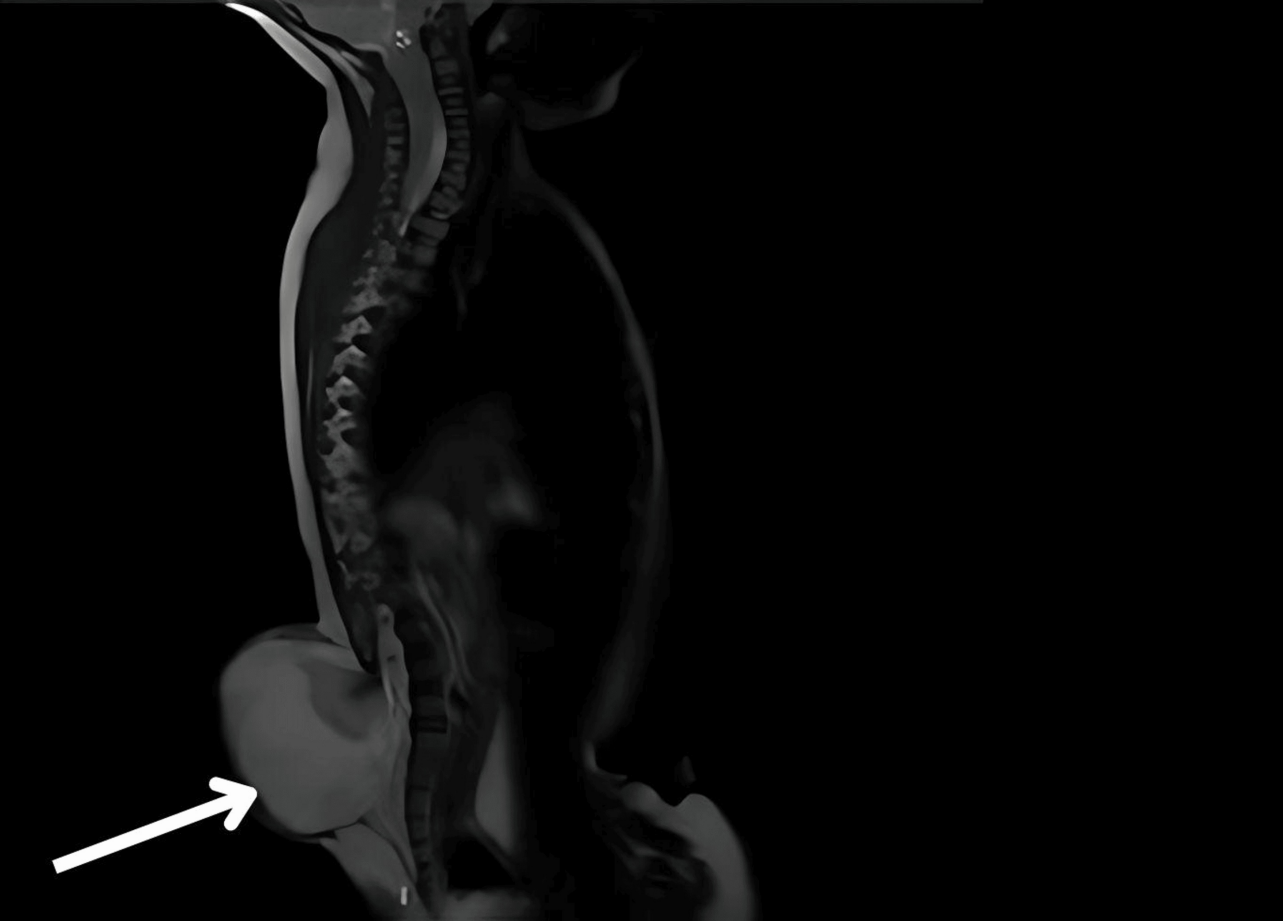
MRI sagittal T2-weighted sequence of the spine in Type 2 Chiari malformation showing absence of the posterior element at the L4 vertebral level with herniation and formation of a fluid-filled sac containing cerebrospinal fluid and neural elements (white arrow). These features are suggestive of myelomeningocele.

**Figure 4:**
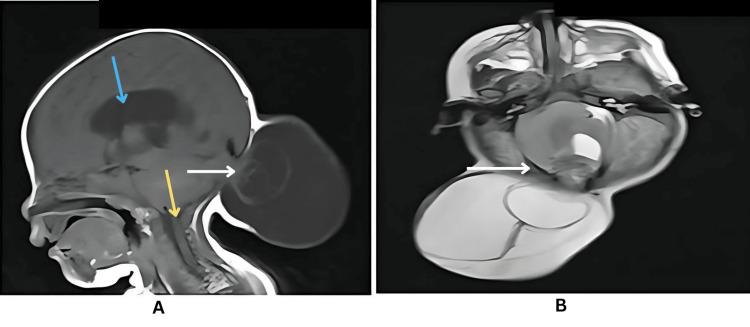
A. MRI sagittal T1-weighted sequence of the brain in Type 3 Chiari malformation showing herniation of posterior fossa contents, including the cerebellar hemisphere and meninges, through a defect (white arrow) in the occipital calvaria. There is downward displacement of the cerebellar tonsil through the foramen magnum (yellow arrow), along with supratentorial ventriculomegaly (blue arrow). These features are suggestive of encephalocele. B. MRI T2-weighted axial sequence of the brain in Type 3 Chiari malformation showing herniation of posterior fossa contents, predominantly the left cerebellar hemisphere and meninges, through a defect in the right occipital calvaria (white arrow). The posterior fossa appears crowded with downward displacement of the right cerebellar tonsil through the foramen magnum, along with supratentorial ventriculomegaly.

**Figure 5:**
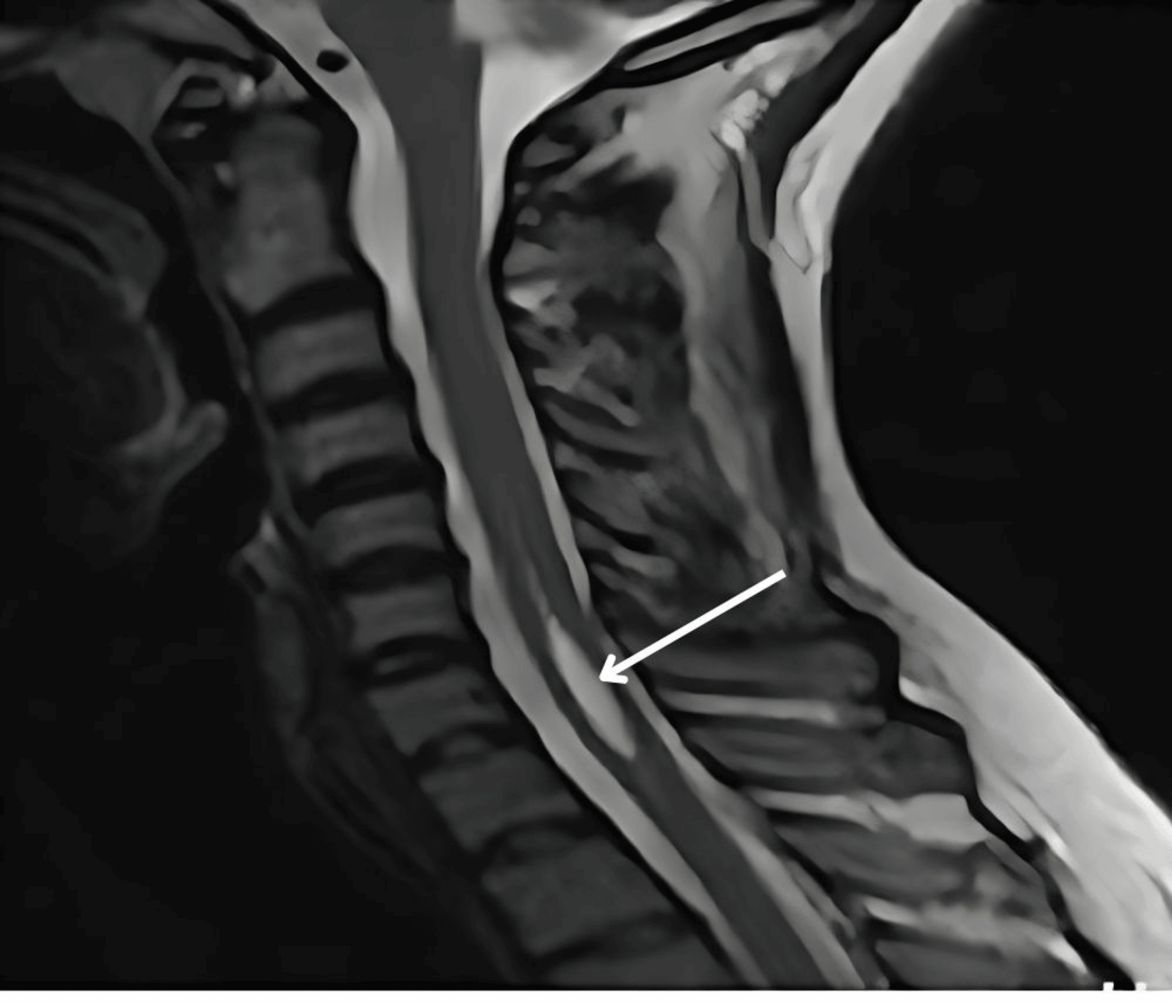
MRI sagittal T2-weighted sequence of the spine in Type 0 Chiari malformation showing a fluid-filled cavity in the cervical region (white arrow), without evidence of herniation of the cerebellar tonsil.

**Figure 6:**
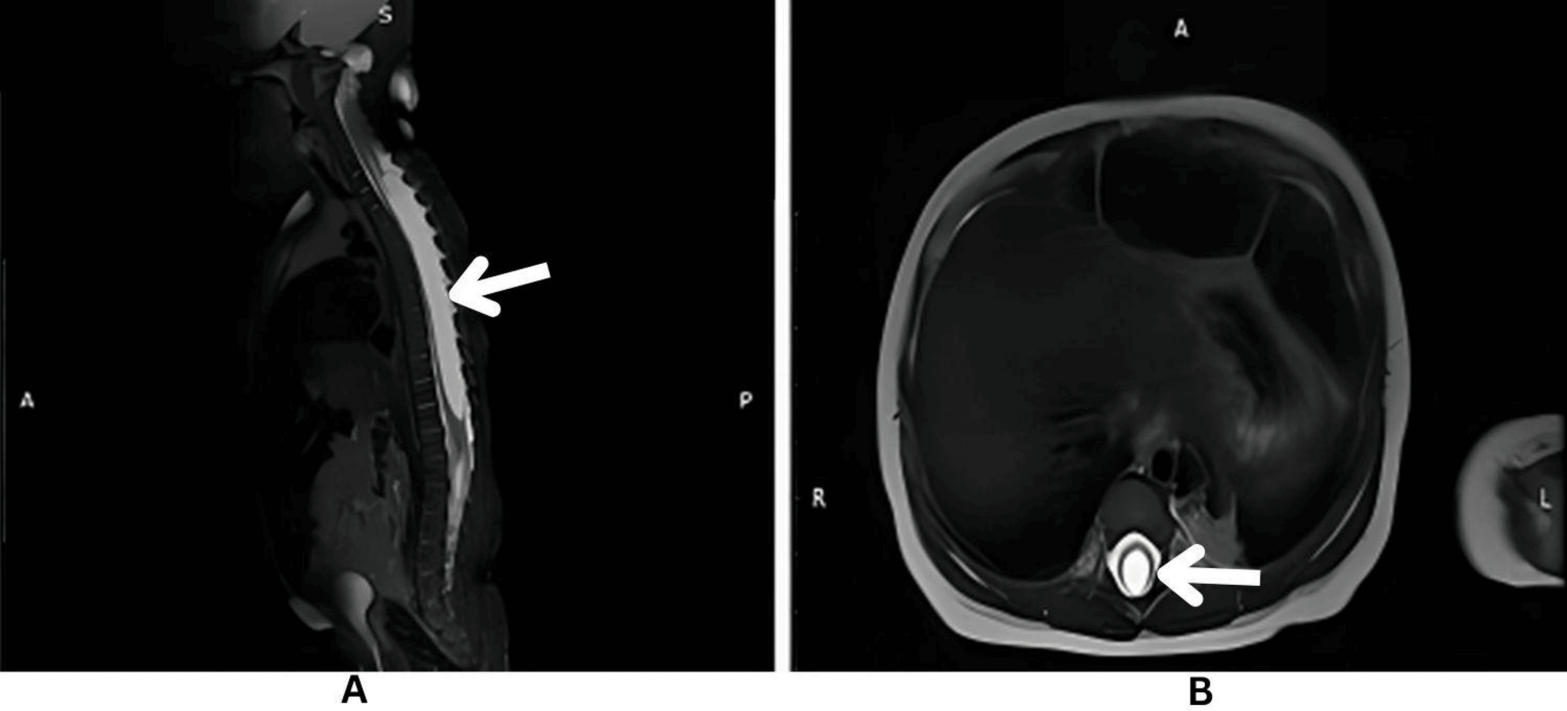
A. MRI sagittal T2 fat-suppressed sequence of the spine showing a fluid-filled cavity (white arrow) in the cervico-thoracic spinal cord. The cavity is continuous and centrally located, and the features are suggestive of a syrinx. B. MRI axial T2-weighted sequence of the spine showing a centrally located fluid-filled cavity (white arrow). The features are suggestive of a syrinx.

Figures 1-5 show MRI (sagittal and axial) images of Type 1, Type 2, Type 3, and Type 0 of Chiari malformation. Figures 6A, 6B show MRI (sagittal and axial) images of a syrinx.

Imaging parameters assessed

Chiari malformation subtypes were classified into Types 0, I, II, and III based on hindbrain morphology. Syrinx characteristics were as follows: presence or absence of syrinx, anatomical level of involvement (cervical, cervico-dorsal, dorsal, dorsolumbar, lumbar, or lumbosacral), and craniocaudal extent (measured in centimeters and categorized as <3 <3 cm or ≥3 cm). Morphology was central vs. eccentric and continuous vs. skip lesions. Posterior fossa features included crowding, tonsillar descent (measured in mm), hydrocephalus, or associated anomalies (myelomeningocele, encephalocele).

Data collection and recording

Clinical and imaging data were prospectively recorded using a structured proforma. Each MRI scan was reviewed independently by two senior radiologists with more than 10 years of experience in pediatric neuroimaging. Discrepancies were resolved by consensus.

Statistical analysis

Data were entered into Microsoft Excel (Microsoft Corp., Redmond, WA) and analyzed using IBM SPSS Statistics version 25.0 (IBM Corp., Armonk, NY, USA). Descriptive statistics were expressed as mean ± standard deviation (SD) for continuous variables and as frequency and percentage for categorical variables. The chi-square test was applied to assess associations between syrinx presence and Chiari subtypes, as well as between syrinx length and clinical features. A p-value of <0.05 was considered statistically significant.

Ethical considerations

All procedures were conducted in accordance with the Declaration of Helsinki (2013 revision). Parental consent and, where appropriate, child assent were obtained. Confidentiality of patient data was strictly maintained, and anonymized identifiers were used for analysis.

## Results

**Figure 7:**
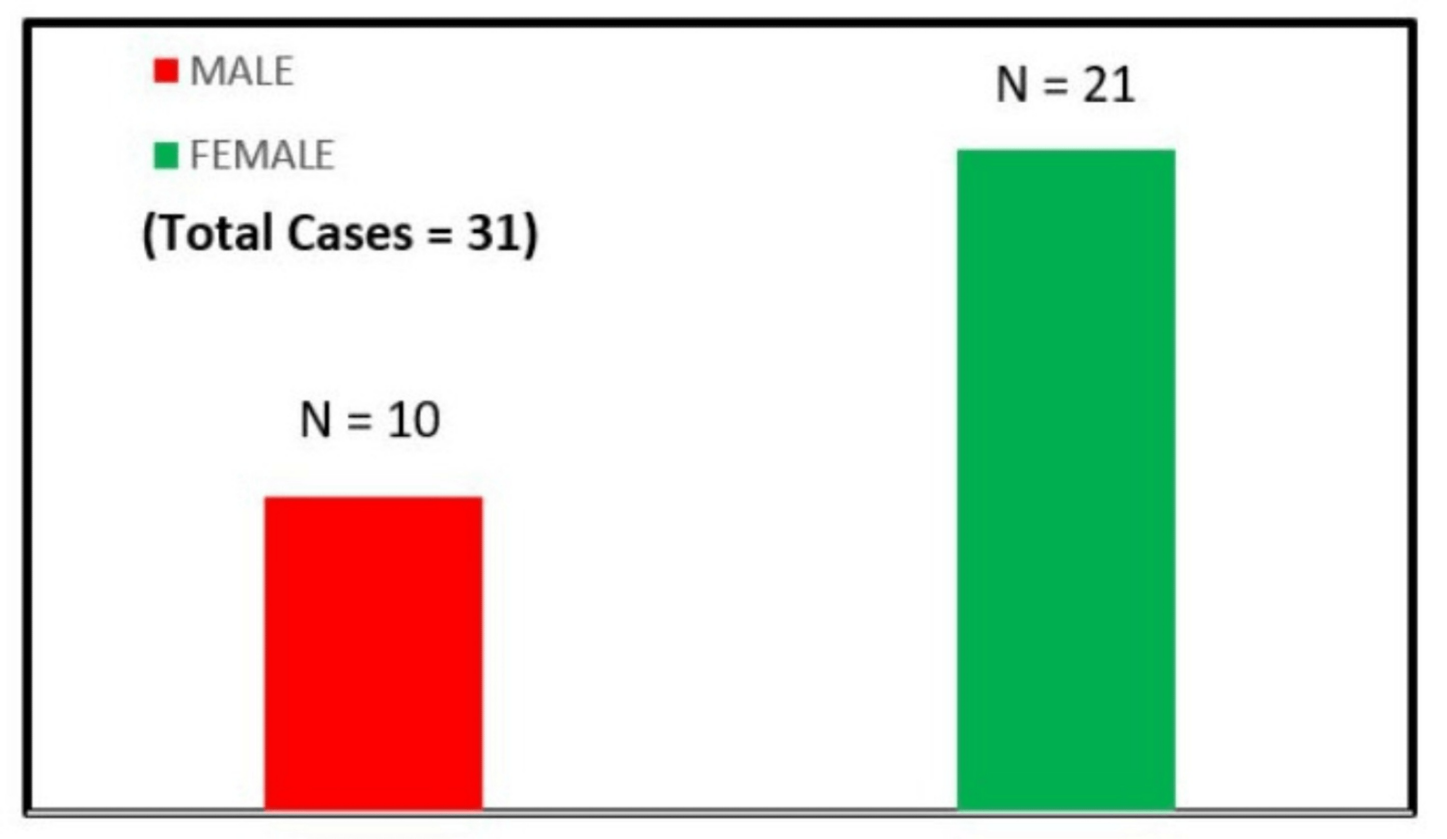
A bar diagram showing sex-wise percentage distribution of patients with Chiari malformation.

A total of 31 pediatric patients aged less than 12 years, presenting with clinical suspicion or an established diagnosis of Chiari malformations, were enrolled between February 2020 and July 2021 at the Institute of Post-Graduate Medical Education and Research and Seth Sukhlal Karnani Memorial Hospital, Kolkata, India. The cohort represented a diverse clinical spectrum, ranging from asymptomatic children referred after incidental detection on neuroimaging to severely symptomatic patients presenting with progressive (35% (N=10)) neurological deficits, giving a female-to-male ratio of nearly 2:1. Of these, 21 patients were female (65%; N=21) and 10 were male. Figure 7 shows the sex distribution of Chiari malformation in the total number of patients in this study.

Age and subtype distribution

**Figure 8:**
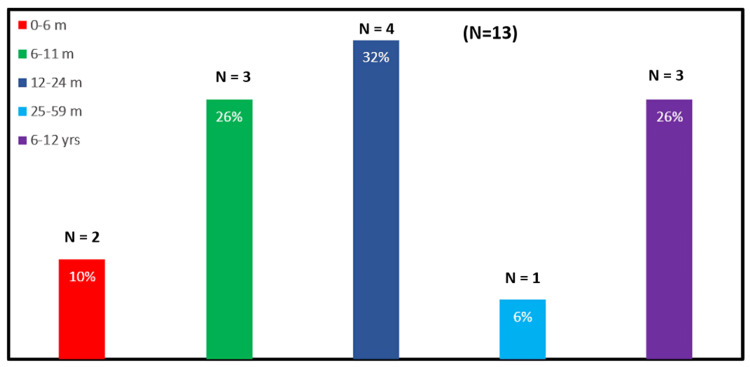
A bar diagram showing age-wise percentage distribution of patients with Chiari malformation

Children with Chiari Type II and III malformations presented much earlier, often in infancy (<18 months), owing to visible structural anomalies such as occipital encephalocele or associated neural tube defects. In contrast, patients with Chiari Type I and Chiari 0 typically came to clinical attention after two years of age, when subtle but progressive neurological symptoms, such as gait disturbance, delayed milestones, or limb weakness, became evident. Figure 8 shows the age distribution of Chiari malformation in the total number of patients in this study.

Clinical spectrum

Presenting complaints varied considerably. A significant proportion of children, 55% (N=17), exhibited LMN signs, including hypotonia, muscle wasting, diminished reflexes, and weakness, often affecting upper limbs. The remaining 45% (N=14) demonstrated UMN involvement with spasticity, exaggerated reflexes, and motor clumsiness. Several patients reported axial symptoms such as scoliosis, chronic headaches, and neck pain. Bulbar features, including swallowing difficulty, recurrent choking, and speech disturbances, were noted in children with more severe hindbrain herniation. Recurrent respiratory infections were seen particularly in Chiari II cases, reflecting brainstem compression and impaired airway protective reflexes. Developmental delays, learning difficulties, and failure to thrive were additional concerns in patients with associated myelomeningocele and hydrocephalus.

Radiological findings

MRI evaluation of the brain and spine was undertaken in all cases using a 3.0 Tesla scanner. Imaging provided clear delineation of the posterior fossa, tonsillar herniation, CSF flow obstruction, and syrinx morphology. Syrinx cavities were present in 43% (N=13) of patients, with the cervico-dorsal region being the most common site (31.4%; N=9), followed closely by cervical (30.4%; N=8) and dorsolumbar (21.7%; N=7) involvement. Less frequent syrinxes were observed in dorsal (8.7%; N=3), lumbar (4.3%; N=2), and lumbosacral (3.3%; N=1) regions. Syrinx length exceeded 3 cm in over half of the patients (55.6%; N=17), reflecting significant cord involvement. Strikingly, all syrinx cavities were central in location and continuous in nature; eccentric and skip lesions, more typical of acquired etiologies such as post-traumatic syringomyelia, were absent in this pediatric Chiari cohort.

A total of 31 (100%) pediatric patients with suspected or confirmed Chiari malformations were included in this prospective observational study conducted over one year. All patients underwent MRI of the brain and spine, and a detailed analysis was performed to characterize spinal cord syrinx with respect to its presence, anatomical distribution, length, morphology, and clinical correlation.

Demographic characteristics

Among the 31 (100%) cases, females predominated, accounting for 65% (N=21) of the cohort, compared to 35% (N=10) males. With regard to age, patients with Chiari Type II and Type III malformations presented significantly earlier, typically within the first 18 months of life, often due to visible abnormalities such as occipital swelling or spinal deformities. In contrast, patients with Chiari Type I and Type 0 malformations presented later, most commonly after two years of age and up to 12 years, usually when neurological symptoms became evident.

Prevalence and association of the syrinx

Syrinx cavities were detected in 43% (N=13) of the total cohort. Statistical analysis revealed no significant independent association between the presence of syrinx and specific Chiari subtypes (p=0.4395). This finding suggests that syrinx formation in Chiari malformations may occur randomly rather than being subtype-dependent.

Anatomical distribution of the syrinx

**Figure 9:**
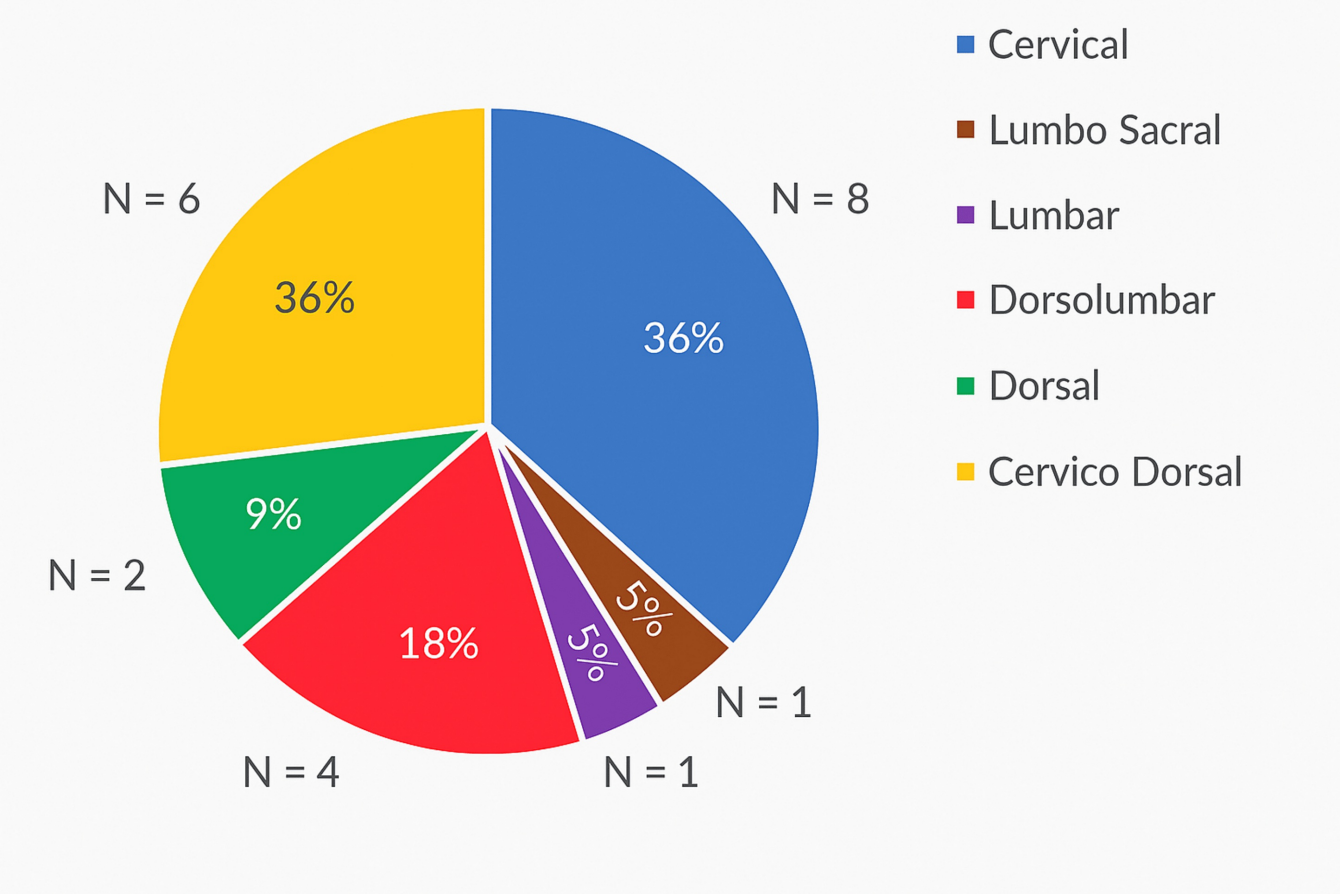
A pie chart showing the level of the syrinx

**Figure 10:**
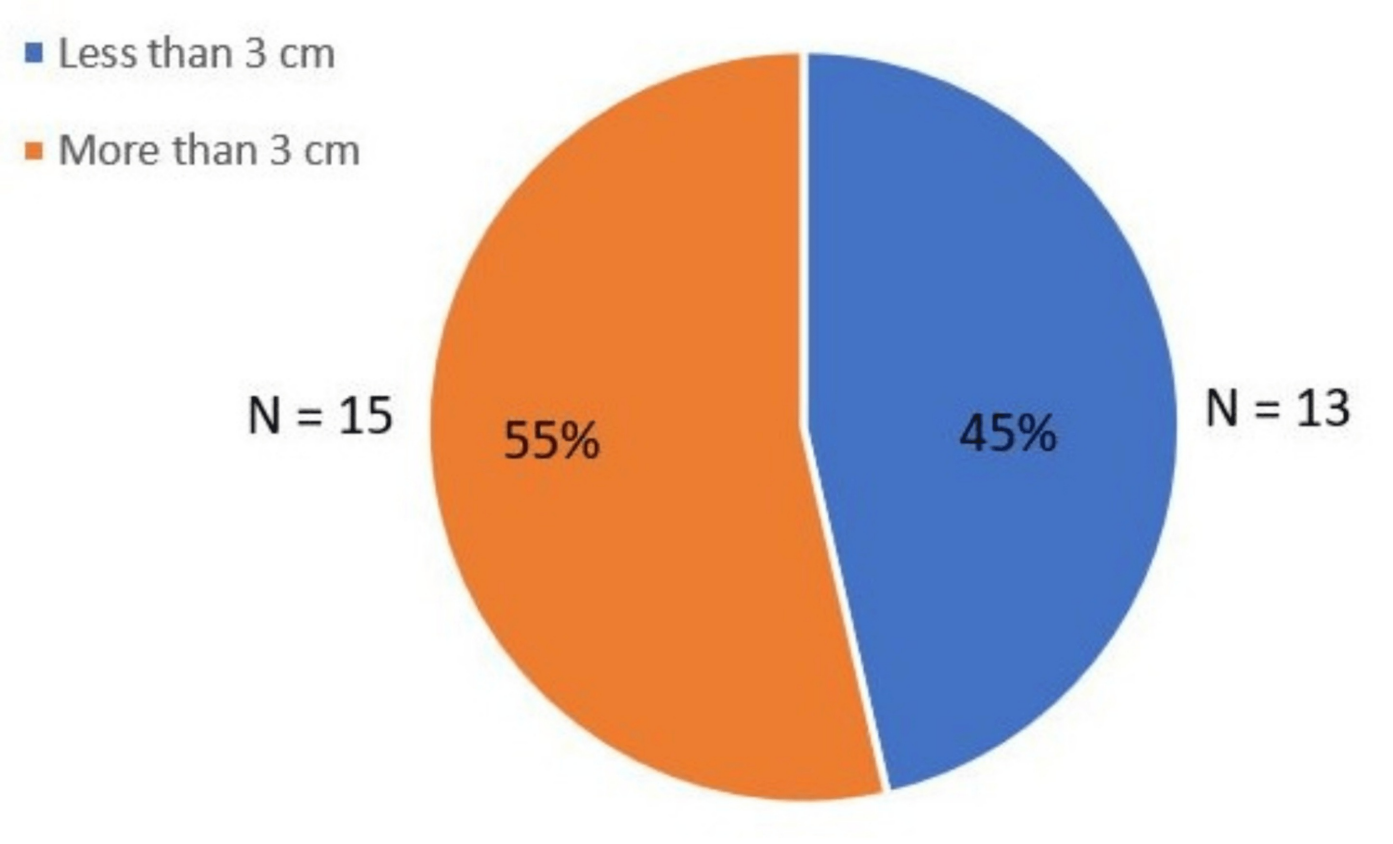
A pie chart showing the length of the syrinx

Analysis of spinal cord levels involved demonstrated that the cervico-dorsal region was the most commonly affected site (31.4%; N=9), followed closely by the cervical segment (30.4%; N=8) and dorsolumbar segment (21.7%; N=7). In comparison, isolated dorsal involvement was observed in 8.7% (N=3) of cases, lumbar in 4.3% (N=2), and lumbosacral in only 3.3% (N=1). These findings highlight a clear predilection for syrinx formation in the cervico-dorsal cord, underscoring the clinical relevance of careful imaging evaluation in this region. Figure 9 shows the level of spinal cord involvement in syrinx in the total number of patients in this study. Figure 10 shows the length of spinal cord involvement in syrinx in the total number of patients in this study.

With respect to craniocaudal extent, 55.6% (N=17) of syrinx cavities extended beyond 3 cm, while 44.4% (N=14) were shorter than 3 cm. The majority of longer syrinx cavities involved multiple contiguous segments, with potential implications for the severity of neurological deficits.

Morphological features of the syrinx

All syrinx cavities detected in this study were centrally located within the spinal cord. No eccentric syrinx formations were observed. Furthermore, every syrinx demonstrated a continuous morphology; no skip-pattern syrinxes were identified. This consistency in morphology may reflect a common underlying pathophysiological mechanism in Chiari-related syringomyelia, in contrast to post-traumatic or tumor-related syrinxes, which often display eccentric or discontinuous patterns.

Clinical correlation

**Figure 11:**
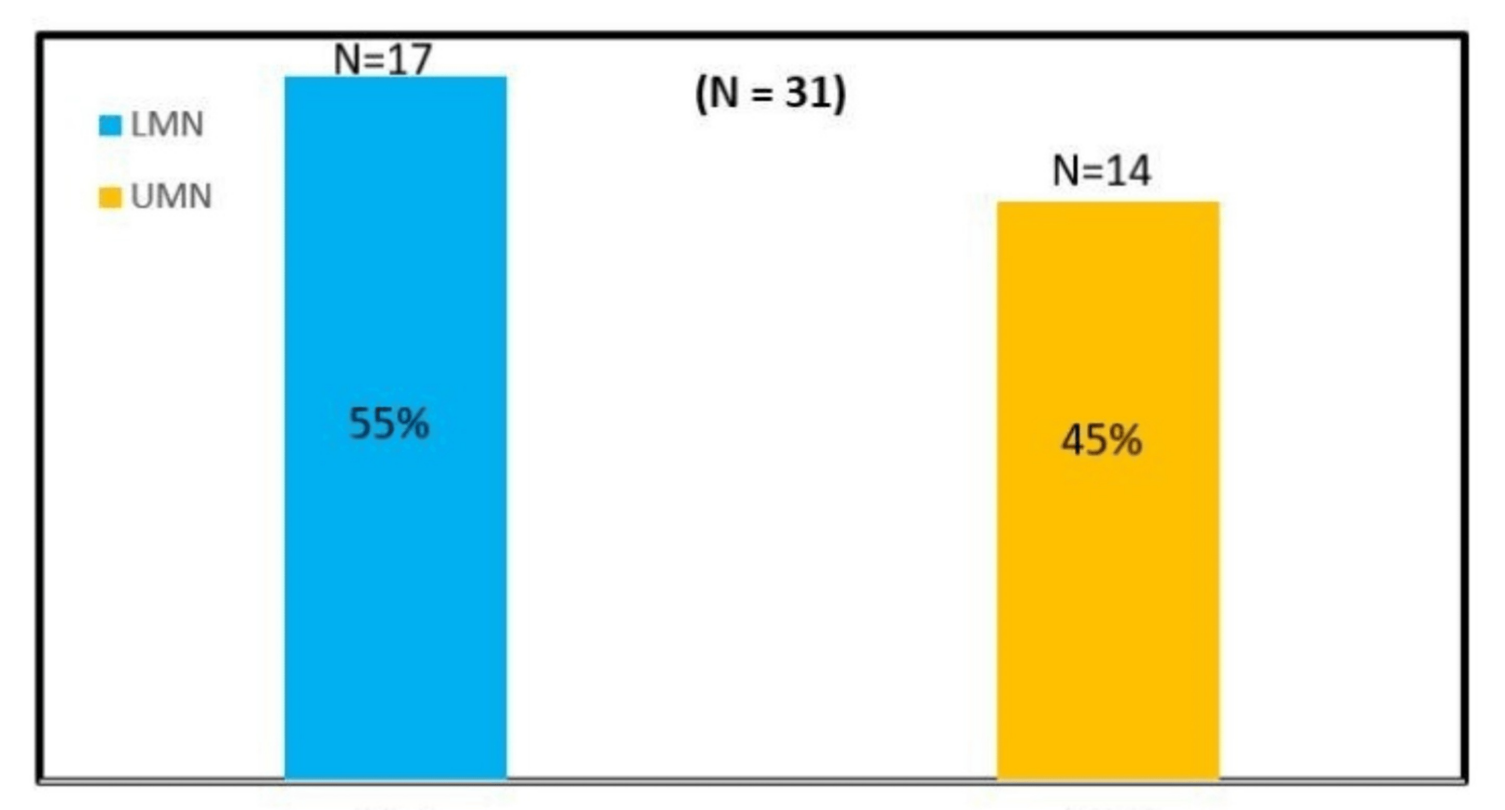
Bar diagram showing percentage of upper motor neuron (UMN) and lower motor neuron (LMN)

Neurological examination revealed that 55% (N=17) of patients demonstrated features of LMN involvement, including muscle weakness, hypotonia, and areflexia, while 45% (N=14) showed signs of UMN dysfunction, such as spasticity and hyperreflexia. The clinical presentation did not show a statistically significant correlation with syrinx level or length, but LMN features were slightly more common, aligning with the predominance of cervico-dorsal syrinx involvement. Figure 11 shows the distribution of LMN and UMN in the total number of patients in this study.

## Discussion

The present study was conducted to evaluate the MRI characteristics of spinal cord syrinx across the spectrum of Chiari malformations in the pediatric population. Particular emphasis was placed on assessing the level, length, and morphology of syrinx cavities, as well as their clinical correlation with UMN and LMN features [12]. The findings of this study provide valuable insights into the complex relationship between Chiari malformations and syringomyelia while also identifying important gaps in existing literature.

In this cohort of 31 (100%) children, Chiari Types II and III malformations were found to present earlier, typically within the first 18 months of life, largely due to the presence of obvious external abnormalities such as swelling at the back of the head or spine. By contrast, patients with Chiari Type I and Type 0 malformations presented later, usually after 24 months, as these subtypes often lack overt physical markers and were only identified once neurological symptoms emerged. This pattern aligns with previous studies, which have noted earlier detection in more severe Chiari variants and delayed recognition in Type I cases due to subtler manifestations [13].

Female predominance was observed in the present study, consistent with earlier reports indicating that Chiari malformations and associated syrinx occur more commonly in females [14]. Clinically, 55% (N=17) of patients presented with LMN features, while 45% (N=14) exhibited UMN signs. This distribution suggests that syrinx-related motor dysfunction does not follow a uniform pattern but is influenced by the level and extent of cord involvement, as also emphasized by Milhorat et al. [15].

A notable finding was that syrinx was not independently associated with any specific Chiari subtype, with statistical analysis revealing a p-value of 0.4395. This implies that the occurrence of syrinx may be more random than subtype-specific, a novel observation that diverges from traditional understanding, where Chiari Type I has been most strongly linked with syringomyelia. This result underscores the complexity of syrinx formation and highlights the need for larger studies to clarify the pathophysiological mechanisms underlying its association with Chiari anomalies.

Regarding anatomical distribution, the cervico-dorsal segment was the most frequently affected region (31.4%; N=9), followed closely by the cervical [30.4% (N=8)] and dorsolumbar (21.7%; N=7) segments. Isolated dorsal, lumbar, and lumbosacral involvement was less common. The predominance of cervico-dorsal syrinx aligns with earlier literature, which identifies this region as particularly vulnerable due to altered CSF flow dynamics at the craniovertebral junction. Importantly, this distribution has direct clinical relevance, as syrinxes at different levels manifest with distinct neurological deficits, influencing both diagnostic evaluation and treatment planning [16].

The majority of syrinxes (55.6%; N=17) extended more than 3 cm in length, a factor often associated with more severe neurological impairment. Interestingly, all syrinxes observed in this study were central in location and continuous in morphology, with no eccentric or skip-pattern cavities identified. This finding contrasts with reports in the literature describing eccentric or discontinuous syrinxes in trauma- or tumor-related cases. The exclusive presence of a central, continuous syrinx in Chiari-associated cases reinforces the concept that these cavities arise primarily from CSF flow obstruction and central canal dynamics rather than parenchymal pathology.

These results contribute meaningfully to bridging key gaps in knowledge. Specifically, hardly any studies have systematically documented the predominant anatomical level of syrinx involvement in Chiari patients or assessed whether syrinx distribution correlates with clinical signs. By establishing cervico-dorsal predominance and identifying random association with Chiari subtypes, the current study provides data that can inform both clinical practice and future research [17].

Nevertheless, certain limitations must be acknowledged. The relatively small sample size restricted the ability to include rarer Chiari subtypes such as Types 1.5, IV, and V, which may have distinct associations with syrinx formation. Similarly, the absence of eccentric or skip-pattern syrinxes in this series may be attributable to sample limitations rather than a true lack of occurrence. Larger, multicentric studies with diverse populations are therefore essential to validate and expand upon these findings.

In conclusion, the present study reinforces the indispensable role of MRI in the evaluation of Chiari malformations and associated syrinx. The findings highlight that syrinxes in pediatric Chiari patients are most often central, continuous, and greater than 3 cm in length, with a strong tendency to involve the cervicodorsal spinal cord. Importantly, syrinx occurrence does not appear to be subtype-specific but rather random across the Chiari spectrum. These insights not only refine the current understanding of Chiari-syrinx relationships but also offer practical implications for early diagnosis, clinical correlation, and management strategies in affected children.

## Conclusions

This study highlights the critical role of MRI in the evaluation of Chiari malformations and associated syrinx in the pediatric population. Syrinxes were most commonly located in the cervico-dorsal spinal cord, typically central in position, continuous in morphology, and extending more than 3 cm in length. Importantly, no significant association was observed between syrinx occurrence and specific Chiari subtypes, suggesting that syrinx formation may be a random rather than subtype-dependent process. Clinically, both UMN and LMN features were noted, reflecting the variable neurological impact of syrinx cavities. While these findings refine our understanding of Chiari-related syringomyelia, the limited sample size points to the need for larger multicentric studies to validate these observations and explore subtype-specific associations. Overall, the study emphasises MRI as an indispensable tool for early diagnosis, clinical correlation, and guiding surgical planning, ultimately contributing to improved outcomes in children with Chiari malformations.
